# Trend Analysis of Respiratory Disease Mortality in the Population Aged 65 and over in Poland: Results from a Registry Study (2000–2022)

**DOI:** 10.3390/arm94010012

**Published:** 2026-02-14

**Authors:** Monika Burzyńska, Małgorzata Pikala

**Affiliations:** 1 Department of Epidemiology and Biostatistics, Public Health Observatory, Medical University of Lodz, Żeligowskiego 7/9, 90-752 Lodz, Poland; malgorzata.pikala@umed.lodz.pl; 2 Successful Ageing Research Unit, Department of Internal Medicine and Geriatric Cardiology, Centre of Postgraduate Medical Education, 01-813 Warszawa, Poland

**Keywords:** chronic lower respiratory diseases, pneumonia and influenza, older adults, mortality, vaccinations, epidemiology

## Abstract

**Highlights:**

**What are the main findings?**

- Mortality from pneumonia and influenza increased markedly among adults aged ≥65 years in Poland across all sex and age subgroups, despite improvements in chronic respiratory disease management.
- Mortality from chronic lower respiratory diseases declined among older men but increased among women, reflecting persistent sex-specific epidemiological differences.

**What are the implications of the main findings?**

- Rising mortality from acute respiratory infections highlights major gaps in vaccination coverage and infection prevention strategies among older adults.
- Strengthening influenza and pneumococcal vaccination uptake and integrating respiratory care within chronic disease management are essential to reduce avoidable respiratory deaths.

**Abstract:**

**Background**: Respiratory diseases remain a major contributor to mortality in Europe, yet national long-term analyses rarely explore sex- and age-specific temporal patterns in detail. Large international datasets provide aggregated estimates but may obscure country-specific trend changes relevant for public health planning. The aim of the study was to assess long-term trends in mortality from chronic lower respiratory diseases (ICD-10: J40–J47) as well as pneumonia and influenza. (ICD-10: J10–J18) in Poland, with particular emphasis on sex- and age-specific trajectories and joinpoint-defined changes over time. **Methods**: All deaths among Polish residents aged ≥65 years were analysed using nationwide mortality registry data. Age-standardised death rates (SDRs) were calculated, and temporal trends were assessed using joinpoint regression models to estimate annual percentage changes (APC) and average annual percentage change (AAPC). **Results**: The proportion of deaths attributable to respiratory diseases increased in both men and women across early (65–74 years) and late (≥75 years) old age. Mortality from chronic lower respiratory diseases declined throughout the study period among men, with the most pronounced reductions observed in the early 2000s, particularly among those aged ≥75 years, while trends among women remained largely stable or showed only gradual declines. In contrast, mortality from pneumonia and influenza rose markedly across all sex and age subgroups, with distinct trend reversals observed after 2008–2009. **Conclusions**: Long-term respiratory mortality trends in Poland exhibit marked sex- and age-specific differences that are not fully captured by aggregated international analyses. These findings highlight the importance of country-level, stratified assessments when interpreting respiratory mortality patterns and underscore the need for caution when relying on single time-point indicators for risk assessment and policy planning.

## 1. Introduction

It is projected that between 2023 and 2100, older adults will constitute an increasingly large proportion of the total population in the European Union. By 2050, the share of individuals aged 65 years and over is expected to rise substantially, reaching nearly 30% of the EU population, compared with 21.3% in 2023. Notably, within the next 36 years, Poland is projected to become the oldest country in Europe [1]. This demographic transition across EU Member States is primarily driven by persistently low fertility rates and advances in medical technologies, which have extended life expectancy and reduced mortality in earlier stages of life.

In response to these demographic changes, governments are increasingly prioritising policies that promote healthy and active ageing, including the development of age-friendly communities that enable older adults to remain active and engaged in social life [2]. Within this context, respiratory diseases represent a particularly important public health challenge. Ageing is strongly associated with a progressive decline in pulmonary function, increased vulnerability to chronic respiratory conditions such as chronic obstructive pulmonary disease, and heightened susceptibility to acute infections, including pneumonia and influenza [3]. As a result, population ageing is likely to amplify the burden of respiratory diseases, both in terms of premature mortality and years of life lost [4]. Consequently, monitoring and preventing respiratory illnesses constitutes a key component of strategies aimed at supporting healthy ageing across Europe.

Respiratory diseases are among the leading causes of morbidity and mortality worldwide, particularly in older age groups. According to Global Burden of Disease (GBD) 2021 estimates, chronic respiratory diseases accounted for more than 4 million deaths globally, with chronic obstructive pulmonary disease (COPD) and lower respiratory infections remaining the predominant contributors [5,6]. Population ageing, especially in Europe, has further intensified the impact of respiratory diseases, as older adults are more susceptible due to age-related declines in lung function, cumulative exposure to risk factors, and a higher prevalence of comorbid conditions [7,8].

In Poland, respiratory diseases rank as the third most common cause of death, following cardiovascular diseases and cancers, accounting for 6.5% of all deaths in 2023. Among individuals aged 65 years and older, respiratory diseases account for nearly twice the proportion of deaths observed in those younger than 65 years [9]. Despite this substantial contribution to mortality in older adults, long-term analyses of respiratory mortality trends in Poland that are simultaneously stratified by sex, age group, and specific cause remain limited.

While international sources such as the Global Burden of Disease study and Eurostat provide valuable comparative estimates of respiratory mortality, they are primarily designed for cross-country comparisons and rely partly on statistical modelling. In contrast, the present study is based on complete national cause-of-death registry data and offers a detailed, long-term analysis of respiratory mortality trends in Poland with simultaneous stratification by sex, age group, and cause-specific categories. The use of joinpoint regression further allows identification of the timing of trend reversals that are not captured in aggregated international datasets. This approach yields Poland-specific epidemiological insights with direct relevance for national prevention strategies and health policy. Addressing this gap is essential for understanding national epidemiological patterns and informing targeted prevention strategies, vaccination policies, and health system planning. Therefore, the aim of this study was to analyse long-term trends in respiratory disease mortality in Poland with a specific focus on sex- and age-specific trajectories and joinpoint-defined changes over time, providing national-level insights complementary to existing international estimates.

## 2. Materials and Methods

The study material consisted of a database including information on all deaths among Polish residents aged 65 years and older between 2000 and 2022, obtained from Statistics Poland. The analysed data include all registered deaths, irrespective of place of death (hospital, home, or other settings), as recorded in the national death registry. The total number of deaths included in the statistical analysis was 6,645,408.

For the present analysis, deaths due to diseases of the respiratory system (ICD-10: J00–J99) were identified, with a focus on the most common groups of causes within this category, namely chronic lower respiratory diseases (J40–J47) and pneumonia and influenza (J10–J18). For the years 2020–2022, deaths due to COVID-19 (U07.1) were also included in a separate analysis. Considering sex- and age-related differences in health problems among older adults, mortality was analysed separately for women and men in two age groups: early old age (65–74 years) and late old age (≥75 years).

To assess changes in the structure of deaths, proportional mortality ratios were calculated for the first and the last year of the study period. Standardised death rates (SDR) were also computed according to the following formula:

$$\mathrm{SDR}=\frac{\sum_{i=1}^{N}\frac{\mathrm{ki}}{\mathrm{pi}}\mathrm{wi}}{\sum_{i=1}^{N}\mathrm{wi}}$$

where ki—number of deaths in the i-th age group,

pi—size of the population in the i-th age group,
wi—weight assigned to the i-th age group, based on the distribution of the standard population,
i—age group number,
N—number of age groups (for five-year age groups within early and late old age, N = 5).

The standardisation procedure was performed using the direct method, in line with the 2012 revision of the European Standard Population [10]. Time trend analysis of SDRs was conducted using Joinpoint regression models and the Joinpoint Regression Program, software developed by the U.S. National Cancer Institute within the Surveillance, Epidemiology and End Results programme [11]. Annual percentage changes (APC) in SDRs were estimated for each segment of the broken lines, as well as average annual percentage changes (AAPC) for the entire study period, together with the corresponding 95% confidence intervals (CI).

The study was approved by Bioethics Committee of the Medical University of Lodz, No. RNN/422/12/KB. Written informed consent for participation was not required for this study in accordance with the national legislation and the institutional requirements.

## 3. Results

Between 2000 and 2022, respiratory diseases remained a major contributor to mortality among adults aged ≥65 years in Poland. The proportion of deaths attributable to respiratory diseases increased over time in both sexes and age groups. Among men aged 65–74 years, this proportion rose from 6.1% in 2000 to 6.9% in 2022, and among men aged ≥75 years from 7.9% to 8.6%. Among women, the increase was more pronounced, from 4.1% to 6.6% in those aged 65–74 years and from 5.1% to 6.5% in those aged ≥75 years.

**Figure 1:**
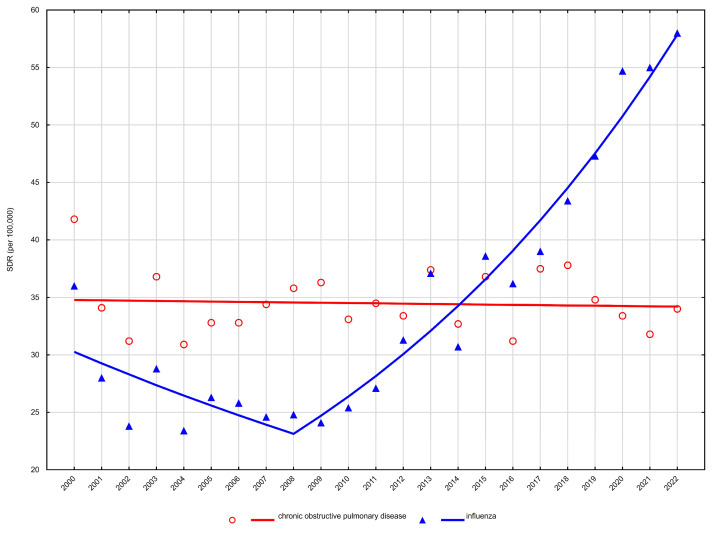
Trends in age-standardised death rates (SDRs) for the most common respiratory disease causes of death among women aged 65–74 years in Poland, 2000–2022.

**Figure 2:**
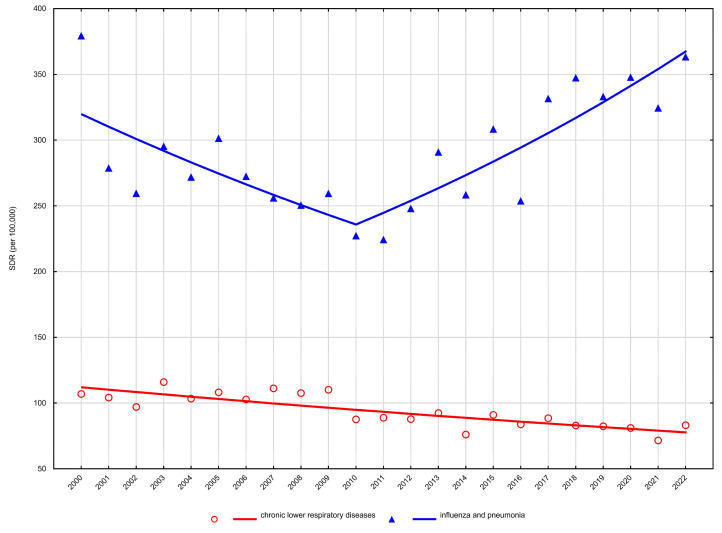
Trends in age-standardised death rates (SDRs) for the most common respiratory disease causes of death among women aged 75 years and over in Poland, 2000–2022.

**Figure 3:**
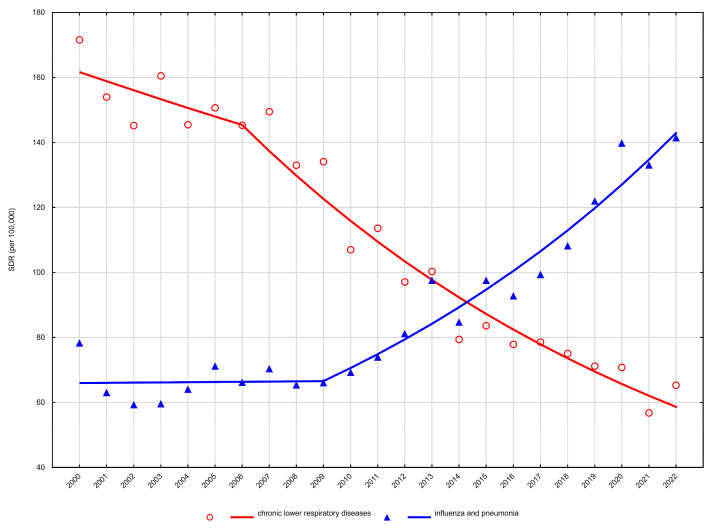
Trends in age-standardised death rates (SDRs) for the most common respiratory disease causes of death among men aged 65–74 years in Poland, 2000–2022.

**Figure 4:**
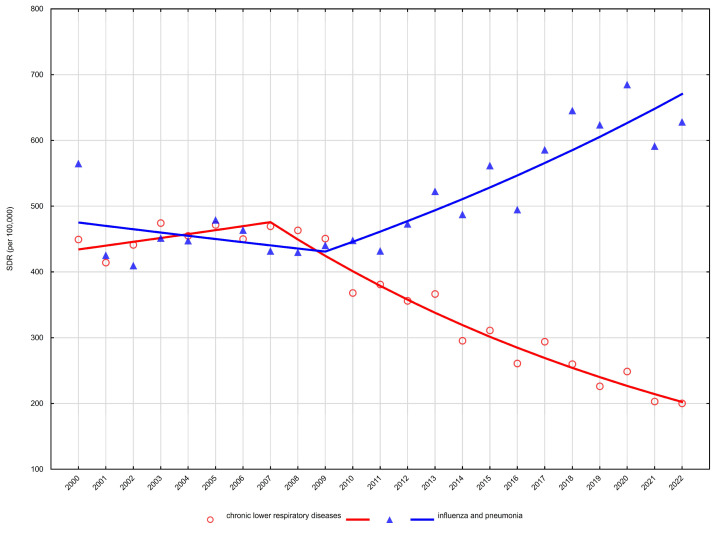
Trends in age-standardised death rates (SDRs) for the most common respiratory disease causes of death among men aged 75 years and over in Poland, 2000–2022.

Age-standardised mortality rates (SDR) showed heterogeneous trends across sex and age groups. Detailed age-standardised mortality rates are presented in Table 1 and Table 2, while temporal trends and joinpoint regression results are summarised in Table 3 and illustrated in Figure 1, Figure 2, Figure 3 and Figure 4.

### 3.1. Chronic Lower Respiratory Diseases

Mortality from chronic lower respiratory diseases demonstrated relatively stable or declining trends throughout the study period, without evidence of sustained post-2008 or post-2010 increases (Table 1 and Table 2).

Among men aged 65–74 years, mortality declined significantly between 2000 and 2022, with a negative average annual percent change (AAPC −2.4%, 95% CI −2.8 to −2.0) (Table 3, Figure 3). A similar pattern was observed in men aged ≥75 years, where mortality decreased steadily over the entire period (AAPC −2.3%, 95% CI −2.8 to −1.9) (Table 3, Figure 4).

In women, trends were more attenuated. Among those aged 65–74 years, mortality from chronic lower respiratory diseases remained largely stable over time (AAPC −0.1%, 95% CI −0.6 to 0.5) (Table 3, Figure 1). In women aged ≥75 years, a modest long-term decline was observed (AAPC −0.8%, 95% CI −1.2 to −0.4), without identifiable joinpoints indicating major temporal shifts (Table 3, Figure 2).

Overall, joinpoint analysis did not identify abrupt reversals or periods of accelerated increase in chronic lower respiratory disease mortality in either sex or age group.

### 3.2. Pneumonia and Influenza

In contrast to chronic respiratory diseases, mortality from influenza and pneumonia exhibited clear and statistically significant temporal changes, characterised by early declines followed by marked increases beginning in the late 2000s.

Among women aged 65–74 years, mortality decreased significantly between 2000 and 2008 (APC −3.3%), after which a strong and sustained increase was observed from 2008 to 2022 (APC +6.8%). This resulted in a significantly positive overall trend across the study period (AAPC +3.0%) (Table 3, Figure 1). A comparable pattern was noted among women aged ≥75 years, with mortality declining between 2000 and 2010 (APC −3.0%), followed by a significant increase from 2010 to 2022 (APC +3.8%). However, the net long-term trend did not reach statistical significance (AAPC +0.6%) (Table 3, Figure 2).

Among men aged 65–74 years, mortality from influenza and pneumonia remained stable between 2000 and 2009, followed by a pronounced increase from 2009 to 2022 (APC +6.1%), yielding a significantly positive AAPC of +3.6% (Table 3, Figure 3). In men aged ≥75 years, a non-significant decline was observed until 2009, after which mortality increased significantly between 2009 and 2022 (APC +3.5%), resulting in a modest but statistically significant long-term increase (AAPC +1.6%) (Table 3, Figure 4).

### 3.3. Sex-Specific Patterns and Timing of Trend Changes

Across all analyses, distinct sex-specific differences in mortality trends and the timing of trend reversals were evident (Table 3). Men experienced earlier and steeper declines in mortality from chronic lower respiratory diseases, particularly from the mid-2000s onward, whereas women showed either stable trends or only modest declines. Conversely, increases in pneumonia and influenza mortality emerged almost simultaneously in both sexes, with joinpoints consistently identified between 2008 and 2009 across age groups.

Notably, in several subgroups—particularly men aged ≥75 years—the overall decline in respiratory mortality masked opposing cause-specific trends, with declining mortality from chronic lower respiratory diseases offset by rising mortality from pneumonia and influenza. These findings highlight the importance of cause-specific analyses for understanding temporal patterns in respiratory mortality among older adults.

### 3.4. COVID-19 Pandemic

In March 2020, the first case of SARS-CoV-2 infection was detected in Poland, marking the onset of the COVID-19 pandemic, which also persisted throughout 2021 and 2022. Overall, between 2020 and 2022, a total of 138,640 deaths due to COVID-19 were recorded among individuals aged 65 years and older in Poland, including 16,900 women in early old age, 50,163 women in late old age, 27,844 men in early old age, and 43,733 men in late old age (Table 4). The highest standardised death rates (SDRs) were observed in 2021 among individuals aged 75 years and older, reaching 2556.1 per 100,000 men and 1431.2 per 100,000 women.

## 4. Discussion

Respiratory diseases remain a major contributor to mortality among older adults in Poland, and the present analysis demonstrates a marked divergence in long-term trends between chronic respiratory conditions and acute respiratory infections. While mortality from chronic lower respiratory diseases has declined or stabilised in some population subgroups—particularly among men—mortality from pneumonia and influenza has increased consistently across all age and sex categories. This pattern mirrors observations from other European countries but appears to be shaped by determinants that are partly specific to the Polish context [5,12].

The most striking finding of this study is the sustained increase in mortality from pneumonia and influenza, even in age groups where total respiratory mortality declined. Among women aged 65–74 years, mortality rates decreased in the early 2000s but reversed sharply after 2008, with a comparable reversal observed among men after 2009. Notably, among men aged ≥75 years, declining mortality from chronic respiratory diseases was accompanied by a continued rise in deaths attributed to pneumonia and influenza. These divergent trends highlight the growing importance of acute respiratory infections as a cause of death in older populations.

Several Poland-specific contextual factors should be considered when interpreting these findings. Cigarette smoking remains the most important modifiable risk factor influencing long-term respiratory mortality trends [6,13,14]. In Poland, smoking prevalence declined sharply among men beginning in the early 1980s, whereas reductions among women occurred later and progressed more slowly, with the highest prevalence observed in female cohorts born between 1940 and 1960 [15,16]. Given the well-established latency period of approximately 20–30 years between smoking exposure and respiratory mortality, these historical patterns are temporally consistent with the observed decline in mortality from chronic lower respiratory diseases among men and the relative stability or increase among women. Nevertheless, the present study does not include individual-level smoking data, and these associations should be interpreted cautiously.

Occupational exposures represent an additional contextual determinant, particularly among men. Historically, male workers in Poland were disproportionately employed in industries associated with high exposure to dusts, fumes, and chemical agents, including mining, metallurgy, heavy manufacturing, and construction. Long-term exposure to such hazards has been linked to increased risks of chronic obstructive pulmonary disease and other chronic respiratory conditions, independent of smoking status [17]. Although individual occupational histories were unavailable in this registry-based analysis, these exposures likely contributed to the sustained male predominance in respiratory mortality observed throughout the study period.

Air pollution plays a significant role in the development of respiratory infections. Numerous epidemiological studies have demonstrated that exposure to ubiquitous pollutants, such as fine particulate matter (PM_2.5_), ozone (O_3_) and nitrogen dioxide (NO_2_), is associated with respiratory diseases and contributes to increased morbidity and mortality [18]. According to the European Environment Agency (EEA) assessment of the burden of disease attributable to air pollution, Poland ranks among the European countries with the highest mortality rates associated with long-term exposure to PM_2.5_ [19]. The WHO guideline recommends that annual PM_2.5_ concentrations should not exceed 5 μg/m^3^; however, in Poland the mean level reached 16.1 μg/m^3^ in 2022, the highest value among EU-27 countries. The largest absolute impacts from long-term PM_2.5_ exposure—exceeding 20,000 attributable deaths—have been estimated for France, Germany, Poland and Italy (in ascending order). Between 2005 and 2023, premature deaths attributable to PM_2.5_ exposure above the WHO air quality guideline level decreased by 57% across the EU-27. In Poland, the decline was slower (−46.1%), resulting in a widening gap between Poland and many other European countries [20]. Because the present study is based on registry mortality data and does not include individual-level exposure information, the role of air pollution should be interpreted as a population-level contextual determinant rather than a direct explanatory factor. Nevertheless, the coexistence of high air-pollution exposure, population ageing, and rising mortality from respiratory infections highlights the importance of integrating environmental health policies with respiratory-disease prevention strategies in Poland.

Beyond behavioural and occupational factors, demographic change plays a critical role in shaping respiratory mortality patterns. The socioeconomic transformation that followed 1989 substantially influenced lifestyle patterns and health-related behaviours in the Polish population [9]. Improvements in population health, supported by advances in medical technologies as well as modern diagnostic and therapeutic methods, contributed to a steady increase in life expectancy. Between 2000 and 2022, life expectancy at birth in Poland increased by 3.8 years among men (from 69.6 to 73.4 years) and by 3.1 years among women (from 78.0 to 81.1 years) [21]. This increase in longevity resulted in a growing proportion of the population surviving into advanced old age, thereby expanding the number of individuals particularly vulnerable to severe infectious respiratory diseases.

Although the present analysis is based on age-standardised mortality rates, increasing life expectancy within older age strata may indirectly contribute to the growing importance of infectious causes of death through the accumulation of chronic conditions, rising multimorbidity, immunosenescence, and frailty [22]. Consequently, part of the observed increase in mortality from pneumonia and influenza likely reflects demographic ageing and improved survival to older ages rather than an isolated increase in infection risk. In addition to demographic and epidemiological determinants, healthcare delivery factors may substantially influence the case-fatality of respiratory infections. Early administration of appropriate antimicrobial therapy is a well-established predictor of survival in pneumonia and sepsis. For example, antibiotic administration within a few hours of hospital admission for community-acquired pneumonia has been associated with reduced mortality in older patients [23], and timely antibiotic treatment in sepsis has been linked with improved short-term survival. At the same time, antimicrobial resistance has become an increasingly important cause of infection-related mortality in Europe [24]. Estimates suggest tens of thousands of deaths annually in Europe attributable to resistant infections, and WHO/ECDC surveillance reports identify antimicrobial resistance as a major regional public-health threat. Guideline-based severity assessment and appropriate level-of-care allocation—including ICU admission when indicated—are also associated with improved outcomes in pneumonia and sepsis care. Moreover, healthcare-associated infections remain a significant contributor to hospital mortality and depend strongly on infection-prevention practices [25]. These mechanisms have also been recognised in national evidence from Poland [26]. Surveillance studies indicate relatively high antibiotic consumption and growing antimicrobial resistance in Poland, while hospital-based analyses document the increasing prevalence of multidrug-resistant organisms [27]. Because the present study lacks detailed clinical management data, these factors should be interpreted as contextual rather than causal explanations of the observed trends.

The temporal inflexion observed after 2008 may partly reflect changes in cause-of-death attribution in multimorbid older adults following the introduction of diagnosis-related group–based hospital financing in Poland. Administrative and diagnostic factors can influence the selection of the underlying cause of death and may therefore have contributed to part of the observed increase in pneumonia and influenza mortality. Moreover, interpretation of respiratory infection trends is further complicated by evolving diagnostic practices over the study period. Earlier years relied predominantly on clinical recognition of pneumonia, whereas access to microbiological confirmation and molecular pathogen detection increased substantially during and after the COVID-19 pandemic. At the same time, limited use of confirmatory imaging and microbiological testing in routine practice may previously have contributed to underrecognition of respiratory infections. Consequently, opposing mechanisms may coexist: historical underdiagnosis in earlier years and improved detection in more recent years. These factors make it difficult to determine whether observed temporal changes reflect true epidemiological shifts or improvements in diagnostic ascertainment. Among specific pathogens, increasing recognition of respiratory syncytial virus (RSV) infection in older adults represents an additional factor potentially influencing mortality attribution. RSV has been shown to cause severe lower respiratory tract infections and substantial mortality in elderly populations, in some settings comparable to influenza. However, systematic testing and reporting in adults were introduced relatively recently, particularly during and after the COVID-19 pandemic. As a result, a proportion of deaths previously classified as unspecified pneumonia may have represented unrecognised RSV infection, leading to underestimation of pathogen-specific respiratory mortality in earlier years.

The contrasting trends observed for chronic lower respiratory diseases further underscore the importance of long-term risk factor modification and advances in disease management. Among men, particularly those aged ≥75 years, mortality from chronic lower respiratory diseases declined steadily, consistent with long-term reductions in smoking prevalence and improvements in pharmacological treatment, pulmonary rehabilitation, and long-term oxygen therapy (3). Among women, however, mortality increased in both analysed age groups, reflecting cohort effects associated with later peaks in smoking uptake and slower declines in smoking prevalence [28].

The observed sex differences in respiratory mortality are consistent with patterns reported across Europe. In most European Union countries, age-standardised mortality rates for chronic respiratory diseases remain substantially higher among men than women, although this gap has narrowed over time [6,29,30]. Differences in smoking history, occupational exposures, disease phenotype, cardiovascular comorbidity burden, and biological susceptibility likely contribute to these disparities [31]. The relative stability of respiratory mortality among women aged <74 years aligns with findings from Western European countries, where declines among men have not been paralleled among women [32,33].

The simultaneous decline in mortality from chronic lower respiratory diseases and increase in mortality from pneumonia and influenza suggests a dual epidemiological pressure. While long-term improvements in chronic disease management may reduce deaths from chronic respiratory conditions, persistent challenges in the prevention and management of acute respiratory infections among older adults remain evident. Poland’s vaccination coverage against influenza and pneumococcal disease remains well below the WHO-recommended target of 75%, with reported coverage of approximately 10–15% among individuals aged ≥65 years [34,35,36,37]. Seasonal variability in circulating influenza virus subtypes may further influence respiratory mortality, although the present study lacks vaccination and virological data to evaluate these effects empirically [38,39,40].

Comparable patterns—declining chronic respiratory mortality alongside increasing mortality from respiratory infections—have been documented in several European countries, indicating that this phenomenon is not unique to Poland [41,42,43]. However, cross-national differences in vaccination uptake, healthcare resources, air quality, and organisation of respiratory care suggest that the underlying drivers may differ between countries, underscoring the importance of national-level analyses.

The COVID-19 pandemic represents an additional complexity in interpreting recent respiratory mortality trends. In the present study, deaths due to COVID-19 were excluded from the analysis of long-term respiratory mortality trends, as their inclusion would have precluded the identification of changes in mortality from respiratory diseases not caused by SARS-CoV-2. Although COVID-19 deaths are coded separately under ICD-10 code U07.1 and should not directly affect mortality trends for diseases coded under J00–J99 [44], evidence from Poland indicates that the pandemic indirectly influenced respiratory mortality patterns. Analyses of excess mortality suggest higher-than-expected deaths from respiratory causes during pandemic waves, likely related to healthcare system overload, delayed diagnosis, postponed procedures, and potential misclassification of causes of death [45]. At the same time, non-pharmaceutical interventions implemented during the pandemic temporarily reduced the circulation of several respiratory pathogens, which may have contributed to a short-term decline in the incidence of non-COVID respiratory infections. These opposing mechanisms highlight the complexity of interpreting respiratory mortality trends during the pandemic period and justify the separate analytical treatment of COVID-19–related deaths.

In summary, the present study demonstrates a growing contribution of acute respiratory infections to mortality among older adults in Poland, occurring alongside declining or stabilising mortality from chronic lower respiratory diseases. These trends reflect a complex interplay of demographic change, historical smoking patterns, occupational exposures, healthcare system factors, and evolving clinical practices. While the registry-based design precludes causal inference, the findings underscore the need for strengthened prevention, timely diagnosis, and evidence-based management of respiratory infections in ageing populations, as well as for continued improvement in the quality and interpretability of cause-of-death data.

### Limitations

An important limitation of this study is its reliance on mortality registry data and on the underlying cause of death recorded on death certificates. In older populations characterised by a high prevalence of multimorbidity, pneumonia and influenza often represent terminal events occurring in the course of chronic diseases rather than the initiating pathological process. Consequently, these conditions may be recorded as the underlying cause of death, potentially leading to an overestimation of pneumonia- and influenza-specific mortality. The observed trends may therefore reflect not only genuine epidemiological changes but also shifts in cause-of-death attribution inherent to registry-based data. In Poland, causes of death are documented as a sequence of events leading to death, and the final ICD-10 code for the underlying cause is assigned centrally by trained physician coders. Despite established quality control procedures, a proportion of non-specific or ill-defined causes of death persists in the registry, which may result in misclassification or underestimation of selected causes. On the other hand, systemic and organisational characteristics of healthcare reporting systems should also be acknowledged. Changes in the hospital reimbursement structure introduced in 2008 may have affected diagnostic coding practices and the classification of underlying causes of death. Temporal variation in documentation and coding behaviour may therefore influence the distribution of recorded causes in administrative and mortality data. Although the present study is based on national mortality registry data rather than hospital discharge records, such factors cannot be fully excluded as a source of classification bias and may have contributed to observed temporal changes in cause-specific mortality patterns. These influences should be interpreted as contextual sources of potential bias rather than evidence of intentional misclassification. Another limitation relates to the incomplete etiological identification of respiratory infections in historical data. Widespread molecular testing for respiratory pathogens, including RSV, became common in Poland only in recent years, particularly after the COVID-19 pandemic. Consequently, earlier mortality data likely underrepresent specific infectious causes and instead classify deaths under broader diagnostic categories such as pneumonia. This may have influenced the distribution of infection-related causes of death over time. The mortality registry does not include individual-level clinical information, such as comorbidities, pharmacotherapy, or the use of advanced respiratory support, precluding adjustment for disease severity or treatment intensity. Accordingly, the present analyses describe population-level mortality patterns rather than individual risk profiles, and causal inference at the individual level is not possible. Nevertheless, owing to its linkage with administrative and legal procedures, the national mortality registry in Poland is considered nearly complete. Despite the above limitations, mortality statistics remain one of the most reliable sources for monitoring long-term population-level mortality trends.

## 5. Conclusions

The present study demonstrates a clear divergence in long-term respiratory mortality patterns in Poland, characterised by declining or stabilising mortality from chronic lower respiratory diseases alongside a persistent increase in deaths attributed to respiratory infections among older adults. These trends appear to reflect the combined effects of population ageing, cohort-related risk exposures, multimorbidity and changing vulnerability to acute illnesses rather than a single dominant determinant. The findings therefore indicate a shift in the structure of respiratory mortality rather than a simple increase in overall respiratory disease burden. From a public health perspective, both long-term prevention of chronic respiratory diseases and improved management and prevention of acute infections remain necessary components of healthcare planning in ageing societies. Clinical outcomes may additionally be influenced by healthcare delivery factors, including timely diagnosis and appropriate organisation of care. Continuous monitoring of cause-specific respiratory mortality is essential for understanding evolving health needs and for adapting preventive and healthcare strategies to changing population structures.

## Figures and Tables

**Table 1:** Standardised death rates (SDR) from the leading causes of death within respiratory diseases among women aged 65–74 years and 75 years and older in Poland, 2000–2022.

Year	Diseases of the Respiratory System (J00–J99)		Chronic Lower Respiratory Diseases (J40–J47)		Influenza and Pneumonia (J10–J18)	
	65–74	75+	65–74	75+	65–74	75+
2000	86.9	526.6	41.8	106.9	36.0	379.4
2001	70.8	419.0	34.1	104.2	28.0	278.8
2002	63.1	395.7	31.2	97.1	23.8	259.5
2003	74.0	450.8	36.8	116.0	28.8	295.3
2004	62.8	411.7	30.9	103.4	23.4	271.9
2005	67.9	445.9	32.8	108.2	26.3	301.4
2006	66.5	412.0	32.8	102.8	25.8	272.5
2007	66.9	401.6	34.4	111.2	24.6	256.0
2008	67.2	389.6	35.8	107.6	24.8	250.6
2009	71.3	408.6	36.3	110.2	24.1	259.4
2010	69.6	357.3	33.1	87.6	25.4	227.3
2011	71.5	349.9	34.5	88.9	27.1	224.4
2012	72.1	362.9	33.4	87.8	31.3	248.0
2013	82.8	410.0	37.4	92.5	37.1	291.0
2014	70.4	359.5	32.7	76.1	30.7	258.4
2015	84.0	438.4	36.8	91.1	38.6	308.4
2016	79.0	383.4	31.2	83.8	36.2	253.8
2017	89.2	464.2	37.5	88.5	39.0	331.6
2018	92.6	466.7	37.8	83.0	43.4	347.4
2019	90.9	445.5	34.8	82.4	47.3	333.1
2020	96.6	457.1	33.4	81.1	54.7	347.8
2021	104.3	457.1	31.8	71.6	55.0	324.4
2022	106.2	508.2	34.0	83.2	58.0	363.4

**Table 2:** Standardised death rates (SDR) from the leading causes of death within respiratory diseases among men aged 65–74 years and 75 years and older in Poland, 2000–2022.

Year	Diseases of the Respiratory System (J00–J99)		Chronic Lower Respiratory Diseases(J40–J47)		Influenza and Pneumonia (J10–J18)	
	65–74	75+	65–74	75+	65–74	75+
2000	278.1	1092.1	171.6	449.4	78.3	564.8
2001	242.2	921.2	154.0	414.2	63.1	425.3
2002	229.4	920.8	145.2	441.2	59.3	409.6
2003	245.3	1001.0	160.5	474.3	59.6	451.5
2004	233.1	966.9	145.5	454.8	64.1	447.5
2005	244.4	1018.3	150.7	471.5	71.2	478.9
2006	233.2	974.9	145.3	449.9	66.2	463.6
2007	241.4	970.6	149.5	469.3	70.4	431.7
2008	218.5	950.8	133.0	463.3	65.4	429.7
2009	227.9	964.0	134.1	450.6	66.1	440.3
2010	205.6	890.5	107.0	368.0	69.3	447.9
2011	212.0	880.1	113.6	381.0	74.0	431.9
2012	197.4	883.9	97.1	356.1	81.2	473.1
2013	216.2	942.0	100.3	366.5	97.6	522.5
2014	180.9	831.9	79.4	295.5	84.7	487.4
2015	201.1	937.4	83.6	311.2	97.6	561.7
2016	196.6	845.7	77.9	261.0	92.8	494.7
2017	204.6	956.8	78.6	294.1	99.4	585.7
2018	206.3	972.8	75.1	259.9	108.2	645.2
2019	213.4	910.5	71.2	226.2	122.0	623.6
2020	230.2	989.8	70.8	248.7	139.8	684.9
2021	226.2	894.5	56.8	203.1	133.1	591.3
2022	242.8	932.0	65.3	200.3	141.5	628.2

**Table 3:** Time trends of standardised death rates (SDR) for the leading causes of death within respiratory diseases among women and men aged 65–74 years and 75 years and older in Poland, 2000–2022—a joinpoint regression analysis.

**Causes of Death**	**Number of Joinpoints**	**Years**	**APC (95% CI)**	**AAPC (95% CI)**
	Women aged 65–74			
Diseases of the respiratory system (J00–J99):	1	2000–2004	−6.6 * (−12.4; −0.4)	1.0 (−0.2; 2.2)
		2004–2022	2.8 * (2.1; 3.5)	
Chronic lower respiratory diseases (J40–J47)	0	2000–2022	−0.1 * (−0.6; 0.4)	
Influenza and pneumonia (J10–J18)	1	2000–2008	−3.3 * (−6.0; −0.5)	3.0 * (1.7; 4.2)
		2008–2022	6.8 * (5.5; 8.1)	
	Women aged 75+			
Diseases of the respiratory system (J00–J99):	1	2000–2011	−2.3 * (−3.6; −0.9)	0.3 (−0.7; 1.2)
		2011–2022	2.8 * (1.4; 4.3)	
Chronic lower respiratory diseases (J40–J47)	0	2000–2022	−1.6 * (−2.1; 5.4)	
Influenza and pneumonia (J10–J18)	1	2000–2010	−3.0 * (−5.0; −0.9)	0.6 (−0.6; 1.9)
		2010–2022	3.8 * (2.1; 5.4)	
	Men aged 65–74			
Diseases of the respiratory system (J00–J99):	1	2000–2015	−1.9 * (−2.5; −1.4)	−0.4 (−1.1; 0.3)
		2015–2022	3.0 * (1.1; 5.0)	
Chronic lower respiratory diseases (J40–J47)	1	2000–2006	−1.8 (−5.3; 1.9)	−4.5 * (−5.5; −3.5)
		2006–2022	−5.5 * (−6.3; −4.7)	
Influenza and pneumonia (J10–J18)	1	2000–2009	0.1 (−2.0; 2.3)	3.6 * (2.5; 4.7)
		2009–2022	6.1 * (4.8; 7.4)	
	Men aged 75+			
Diseases of the respiratory system (J00–J99):	0	2000–2022	−0.4 * (−0.7; 0.0)	
Chronic lower respiratory diseases (J40–J47)	1	2000–2007	1.3 (−1.1; 3.8)	−3.4 * (−4.2; −2.6)
		2007–2022	−5.5 * (−6.3; −4.8)	
Influenza and pneumonia (J10–J18)	1	2000–2009	−1.1 (−3.2; 1.1)	1.6 * (0.5; 2.7)
		2009–2022	3.5 * (2.2; 4.8)	

* *p* < 0.05.

**Table 4:** Number of deaths and standardised death rates (SDR) from COVID-19 among women and men aged 65–74 years and 75 years and older in Poland, 2020–2022.

Year	Women				Men			
	65–74		75+		65–74		75+	
	Number of Deaths	SDR	Number of Deaths	SDR	Number of Deaths	SDR	Number of Deaths	SDR
2020	3896	159.4	11,590	607.3	7426	396.4	11,797	1301.7
2021	10,358	411.2	27,479	1431.2	16,378	846.9	23,137	2556.1
2022	2646	103.7	11,094	567.2	4040	206.8	8799	988.9

## Data Availability

The original contributions presented in this study are included in the article. Further inquiries can be directed to the corresponding author.

## Abbreviations

The following abbreviations are used in this manuscript:

SDR	Standardised Death Rates
APC	Annual Percentage Change
AAPC	Average Annual Percentage Change
